# Enhanced therapeutic effects of umbilical cord mesenchymal stem cells after prolonged treatment for HBV-related liver failure and liver cirrhosis

**DOI:** 10.1186/s13287-020-01787-4

**Published:** 2020-07-10

**Authors:** Yifan Jia, Xin Shu, Xiaoan Yang, Haixia Sun, Huijuan Cao, Hong Cao, Ka Zhang, Qihuan Xu, Gang Li, Yang Yang

**Affiliations:** 1 Department of Infectious Diseases, The Third Affiliated Hospital of Sun Yat-Sen University, Guangzhou, People’s Republic of China; 2 Department of Liver Surgery and Liver Transplantation, The Third Affiliated Hospital of Sun Yat-Sen University, Guangzhou, People’s Republic of China

**Keywords:** Umbilical cord mesenchymal stem cell transplantation, Hepatitis B virus, Liver failure, Liver cirrhosis, Therapeutic effects

## Abstract

**Background:**

Umbilical cord mesenchymal stem cells (UCMSCs) have been demonstrated to have good therapeutic effects in the treatment of HBV-related liver diseases. However, the therapeutic effect of UCMSCs on HBV-related liver failure and liver cirrhosis and the variations in the efficacy of UCMSCs after different treatment courses remain poorly understood. Therefore, this study was designed to answer these two questions.

**Methods:**

This was an observational study that retrospectively considered a 3-year period during which 513 patients who received stem cell infusion and met the criteria of hepatic failure and liver cirrhosis were identified from the databases of the Third Affiliated Hospital of Sun Yat-sen University. The eligible patients were categorized into the liver failure group and liver cirrhosis group. The two groups were divided into different subgroups according to the duration of stem cell therapy. In the liver failure group, group A received more than 4 weeks and group B received less than 4 weeks of stem cell therapy. In the liver cirrhosis group, patients who received more than 4 weeks of stem cell therapy belonged to group C, and the patients in group D received less than 4 weeks of stem cell therapy. The patients were followed up for 24 weeks. The demographics, clinical characteristics, biochemical factors, and model for end-stage liver disease (MELD) scores were recorded and compared among different groups.

**Results:**

A total of 64 patients met the criteria for liver failure, and 59 patients met the criteria for liver cirrhosis. After UCMSC treatment, the levels of alanine aminotransferase (ALT), glutamic-oxaloacetic transaminase (AST), and total bilirubin (TBIL) at all postbaseline time points were significantly lower than those at baseline in the liver failure group and liver cirrhosis group; the prothrombin activity (PTA) and MELD scores gradually improved in only the liver failure group. Four weeks after UCMSC treatment, patients who received prolonged treatment with UCMSCs had a larger decrease in TBIL levels than patients who terminated treatment with UCMSCs. After more than 4 weeks of UCMSC treatment, there were no statistically significant differences in the changes in ALT, AST, TBIL, and PTA values and MELD scores between patients with liver failure who received prolonged treatment with UCMSCs and patients with liver cirrhosis who received prolonged treatment with UCMSCs at any time point. However, the median decrease and cumulative decrease in the TBIL level of patients with liver failure with a standard 4-week treatment course were larger than those of patients with liver cirrhosis with a standard 4-week treatment course.

**Conclusion:**

Peripheral infusion of UCMSCs showed good therapeutic effects for HBV-related liver failure and liver cirrhosis. Prolonging the treatment course can increase the curative effect of UCMSCs for end-stage liver disease, especially for patients with cirrhosis.

## Introduction

Liver failure and cirrhosis are the results of chronic liver damage caused by many factors, including alcohol, drugs, and hepatitis virus (HBV, HCV, etc.), among which hepatitis B virus (HBV) infection is the most common cause of liver failure and cirrhosis, with high mortality and a large economic burden [1]. HBV-related end-stage liver disease mainly includes liver failure and decompensated cirrhosis. Due to its rapid progression and poor prognosis, liver transplantation is considered the most effective treatment for patients with HBV-related end-stage liver disease [2]. However, the application of liver transplantation is limited by the shortage of donor livers, the risks of transplantation, and the long-term use of immunosuppressants after transplantation [3]. Therefore, the majority of gastroenterologists/hepatologists have been looking for new treatment methods to treat patients with HBV-related end-stage liver disease.

Twenty years ago, Theise et al. [4] reported that Y chromosome-positive hepatocyte-like cells were present in the livers of women who had received allogenic bone marrow transplantations from male donors and concluded that pluripotent stem cells may exist among bone marrow cells. Stem cell therapy has been applied in the treatment of end-stage liver disease, and its clinical efficiency is satisfactory [5]. Many basic and clinical studies have provided evidence that MSCs are safe and effective in the treatment of liver failure and cirrhosis. With advancements in research, scholars have also proposed a series of hypotheses related to the factors that influence MSCs during the treatment of liver failure and cirrhosis, such as the type of MSCs, the time of infusion, the method of infusion, and the dosage of infusion [6, 7]. Previous research in our department showed that allogeneic bone marrow-derived MSCs are safe and effective for patients with HBV-related acute-on-chronic liver failure (ACLF) [8, 9]. However, autologous mesenchymal stem cells often lead to delayed treatment due to the time needed for culture. Ethical issues and uncertainty about the possibility of malignant differentiation in vivo also limit the clinical application of these cells. Fortunately, allogeneic mesenchymal stem cells can overcome this limitation and can be widely used in the clinic as “ready-made” immune-privileged therapy agents. Embryonic stem cells (ESCs) and induced pluripotent stem cells (IPSs) have been shown to be the cells most capable of producing a large number of functional liver-like cells (HLCs) in mice and humans [10]. MSCs from the umbilical cord performed as well as those from the bone marrow and better than other types of adult stem cells, although no study showed complete and sustainable performance in the outcome measures [11].

Nevertheless, almost all studies have focused on only liver failure or cirrhosis. Is there a difference in the efficacy of umbilical cord mesenchymal stem cells (UCMSCs) between patients with HBV-related liver failure and patients with liver cirrhosis in the real-world setting? Is there a difference in the efficacy of UCMSCs with different treatment courses in patients with HBV-related liver failure or cirrhosis? We conducted this research to try to answer these two questions.

## Methods

### Study population

The data of patients with HBV-related liver failure or liver cirrhosis who received UCMSCs at the Third Affiliated Hospital of Sun Yat-sen University between February 2014 and December 2015 were collected. All study procedures adhered to the tenets of the Declaration of Helsinki, and informed consent was obtained from all patients. The study was approved by the Human Ethics Committee of The Third Affiliated Hospital of Sun Yat-sen University, Guangzhou, China. Patients who met the following criteria were deemed eligible for enrollment in this study: (A) HBsAg positive for more than 6 months, (B) met the 2009 APASL diagnostic criteria for hepatitis B liver failure and cirrhosis [12]; and (C) aged between 16 and 60 years. Patients were excluded for the following reasons: (A) severe complications, such as esophageal and gastric vein rupture and hemorrhage, and septicemia, in the last 2months; (B) other autoimmune diseases; (C) infection with other hepatitis viruses; (D) impaired function of other important organs; (E) pregnant or lactating; (F) human liver or liver transplantation; (G) alcoholic liver disease; and (H) liver cancer.

### Study design

All patients with HBV-related end-stage liver disease who were treated with UCMSCs participated in the study. According to the diagnosis, the patients were divided into two groups: the liver failure group and the liver cirrhosis group. The liver failure group was then categorized into group A and group B. Group A included patients with liver failure who were treated with UCMSCs for more than 4 weeks; group B included patients with liver failure who received UCMSCs for less than or equal to 4 weeks. In the liver cirrhosis group, patients who received more than 4 weeks of UCMSC therapy belonged to group C, and group D included patients with liver cirrhosis who were treated with UCMSCs for less than or equal to 4 weeks. The patients were followed up for 24 weeks. Data from all patients were collected at baseline and at 1, 4, 12, and 24 weeks after therapy. All patients in the study received standard clinical treatments (including albumin supplementation, coagulation correction, S-adenosylmethionine infusion, antiviral treatment, and necessary anti-infective treatment) for approximately 1 week before stem cell infusion. One week later, all patients received the appropriate dose of stem cells after being provided the relevant information about stem cell treatment and signing the informed consent form. The levels of alanine aminotransferase (ALT), glutamic-oxaloacetic transaminase (AST), total bilirubin (TBIL), and prothrombin activity (PTA) and the model for end-stage liver disease (MELD) scores were analyzed to comprehensively evaluate the infusion effects of different doses of stem cells.

### UCMSC preparation and transfusion

The processing of the umbilical cords and preparation of UCMSCs were performed at the GMP Stem Cell Laboratory Facility of the Biotherapy Center of The Third Affiliated Hospital of Sun Yat-sen University, Guangzhou, China. Fresh human umbilical cords were obtained after birth and collected in phosphate-buffered saline at 4 °C with consent from the parents. The specific details of the isolation, culture, and characterization of UCMSCs were described in previous research [13]. Bacterial, fungal, and viral monitoring (including hepatitis B virus [HBV], hepatitis C virus [HCV], human immunodeficiency virus [HIV], and cytomegalovirus) was performed for all umbilical cords and prior to injection for quality control. Patients receiving less than or equal to 4 UCMSC transfusions received them at 1, 2, 3, and 4 weeks after recruitment. Patients receiving more than 4 UCMSC transfusions received them at 1, 2, 3, 4, 5, 6, 7, and 8 weeks after recruitment. As reported in previous research (ClinicalTrials.gov, NCT02223897, [14]), approximately 1.0 × 10^6^ UCMSCs per kilogram of body weight suspended in 100 mL of normal saline solution were infused intravenously through a vein in the forearm for each treatment. The patients were discharged after 1 day of observation without any adverse events.

### Statistical analysis

SPSS software (version 22.0; SPSS, Inc., Chicago, IL) was used to perform the statistical analyses. The Shapiro-Wilk test for normality was performed. Normally distributed variables were analyzed with ANOVA, while nonnormally distributed variables were analyzed with nonparametric tests. Continuous variables were expressed as the mean ± standard deviation (SD) or median (interquartile) depending on the result of normality testing and were analyzed using a *t* test or the Wilcoxon test, as appropriate. Sex was expressed as the number of patients (percentage) and tested with the χ2 test or Fisher’s exact test, as appropriate. All analyses were performed as two-sided tests with a 0.05 level of significance.

## Results

### Patient characteristics

A total of 513 patients signed informed consent forms and received UCMSC treatment. According to the diagnostic criteria and matching treatment and medication records, only 123 patients were enrolled and eligible for efficacy analysis in the present study. Of these patients, 64 were included in the liver failure group and 59 in the cirrhosis group. The patients were divided into four groups: prolonged treatment liver failure (group A, *N* = 37), standard treatment liver failure (group B, *N* = 27), prolonged treatment liver cirrhosis (group C, *N* = 28), and standard treatment liver cirrhosis (group D, *N* = 31). The baseline characteristics of the 123 patients in this study are shown in Tables 1 and 2. These variables were generally similar between group A and group B, and no significant differences in baseline characteristics were observed between group C and group D (Table 1). However, the difference between group A and group C was statistically significant; similarly, the difference between group B and group D was statistically significant (Table 2).

**Table 1:** Patient demographics and baseline characteristics between group A and group B and between group C and group D

Factors	Liver failure			Liver cirrhosis		
	Group A	Group B	***P*** value	Group C	Group D	***P*** value
Sex (M/F)	34/3	22/5	0.266	26/5	23/5	1.000
Age (years)	40 (35–46.5)	42 (33–50)	0.581	47.5 (37.5–56.5)	44 (35–60)	0.665
ALT (U/L)	88 (46.5–312)	77 (46–336)	0.703	48 (23.25–75.75)	42.5 (25.25–89)	0.716
AST (U/L)	109 (68–173.5)	103 (68–147)	0.760	73 (45.25–131.5)	65 (47.5–92)	0.268
TBIL (mmol/L)	486 (346–550.5)	381 (219–495)	0.053	415 (85.25–488.5)	149 (75.7–306.75)	0.027
PTA	35 (25.5–40.5)	34 (31–47)	0.438	40.5 (29.75–53)	44.5 (34.75–59.25)	0.161
MELD	78 (76.5–81)	77 (75–82)	0.411	76 (73–78)	72 (67.75–76)	0.005

*ALT* alanine aminotransferase, *AST* glutamic-oxaloacetic transaminase, *TBIL* total bilirubin, *PTA* prothrombin time activity, *MELD* model for end-stage liver disease

**Table 2:** Patient demographics and baseline characteristics between group A and group C and between group B and group D

Factors	> 4 weeks			≤ 4 weeks		
	Group A	Group C	***P*** value	Group B	Group D	***P*** value
Sex (M/F)	34/3	23/5	0.275	22/5	26/5	1.000
Age (years)	40 (35–46.5)	47.5 (37.5–56.5)	0.035	42 (33–50)	44 (35–60)	0.435
ALT (U/L)	88 (46.5–312)	48 (23.25–75.75)	0.004	77 (46–336)	42.5 (25.25–89)	0.005
AST (U/L)	109 (68–173.5)	73 (45.25–131.5)	0.054	103 (68–147)	65 (47.5–92)	0.002
TBIL (mmol/L)	486 (346–550.5)	415 (85.25–488.5)	0.043	381 (219–495)	149 (75.75–306.75)	0.000
PTA	35 (25.5–40.5)	40.5 (29.75–53)	0.033	34 (31–47)	44.5 (34.75–59.25)	0.006
MELD	78 (76.5–81)	76 (73–78)	0.006	77 (75–82)	72 (67.75–76)	0.000

*ALT* alanine aminotransferase, *AST* glutamic-oxaloacetic transaminase, *TBIL* total bilirubin, *PTA* prothrombin time activity, *MELD* model for end-stage liver disease

### Therapeutic effect of UCMSCs in the liver failure group and liver cirrhosis group

**Fig. 1:**
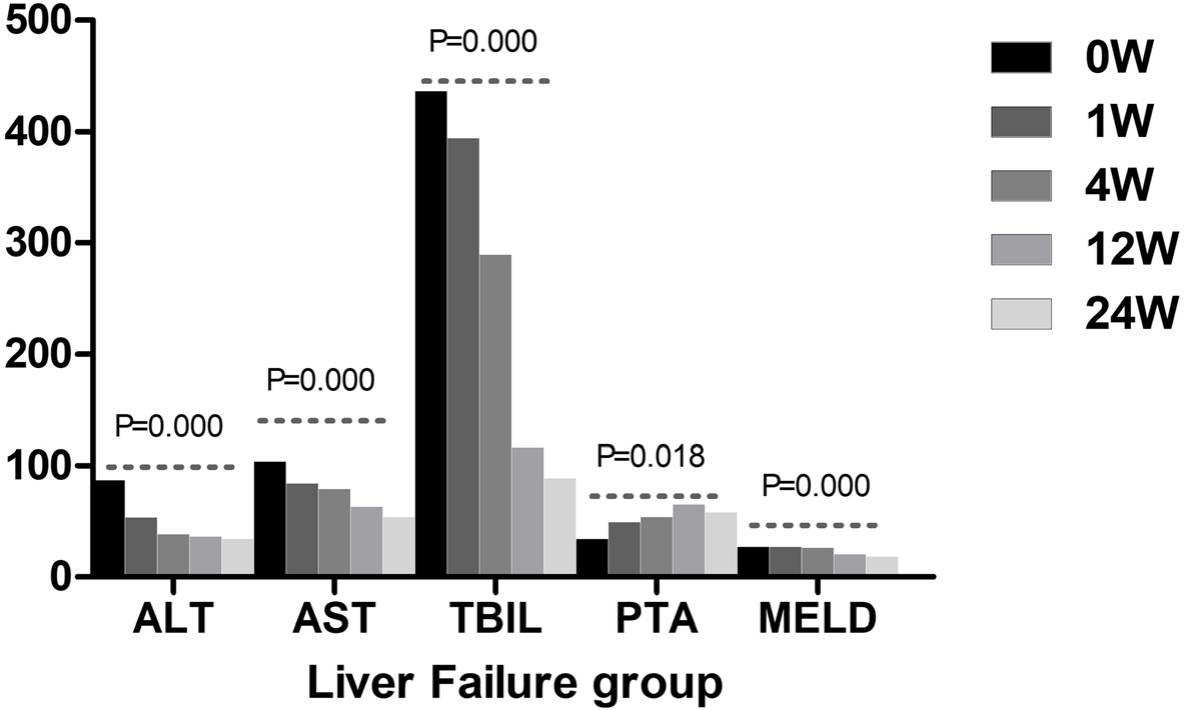
Comparison of the therapeutic effect of UCMSCs among different observation weeks in the liver failure group. ALT, alanine aminotransferase; AST, glutamic-oxaloacetic transaminase; TBIL, total bilirubin; PTA, prothrombin time activity; MELD, model for end-stage liver disease

**Fig. 2:**
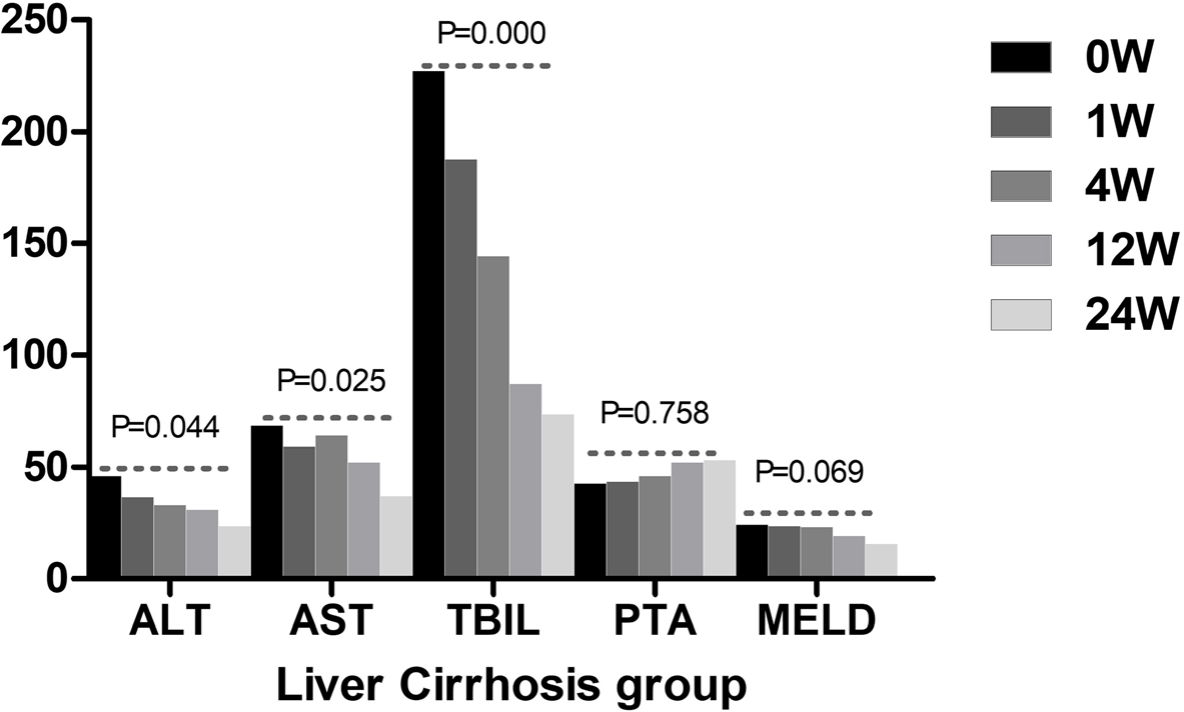
Comparison of the therapeutic effect of UCMSCs among different observation weeks in the liver cirrhosis group. ALT, alanine aminotransferase; AST, glutamic-oxaloacetic transaminase; TBIL, total bilirubin; PTA, prothrombin time activity; MELD, model for end-stage liver disease

**Fig. 3:**
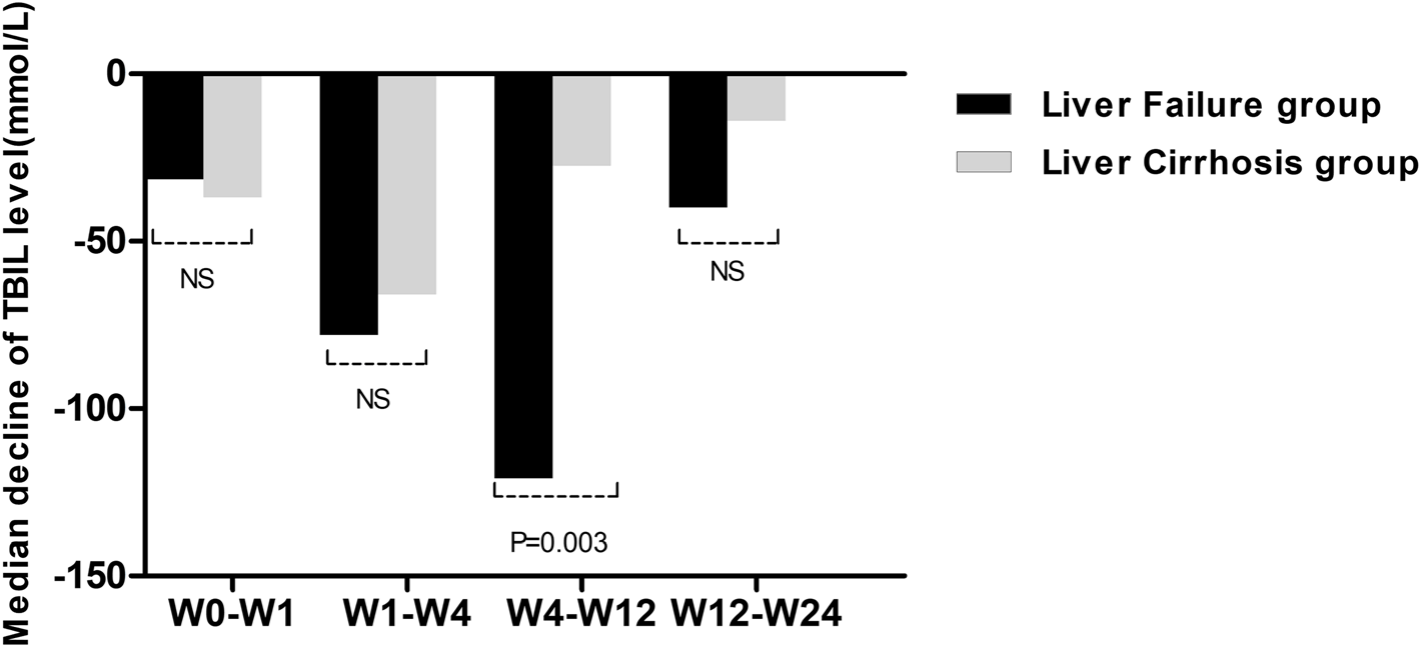
Comparison of the median decreases in TBIL levels between the liver failure group and liver cirrhosis group at different time points. TBIL, total bilirubin

The therapeutic effect of UCMSCs varied based on the week of observation in the liver failure group and liver cirrhosis group. The levels of ALT, AST, and TBIL and the MELD score at all postbaseline time points were significantly lower than those at baseline in the liver failure group, and the value of PTA gradually increased after UCMSC treatment in the liver failure group (Fig. 1). In the liver cirrhosis group, the levels of ALT, AST, and TBIL progressively decreased after UCMSC treatment. However, no significant differences in PTA values and MELD scores were observed among all observation weeks (Fig. 2). The decrease in TBIL varied significantly according to the treatment regimen. The median serum TBIL decrease in the liver failure group was significantly larger than that in the liver cirrhosis group from week 4 to week 12 (*P* = 0.003, Fig. 3). However, no apparent difference was found at the other three time points (Fig. 3). Similarly, no statistically significant changes in the levels of ALT, and AST, PTA values or MELD scores were found between the two groups at any of the four time points.

### Comparative analysis of the therapeutic effect of UCMSCs between group A and group B

**Fig. 4:**
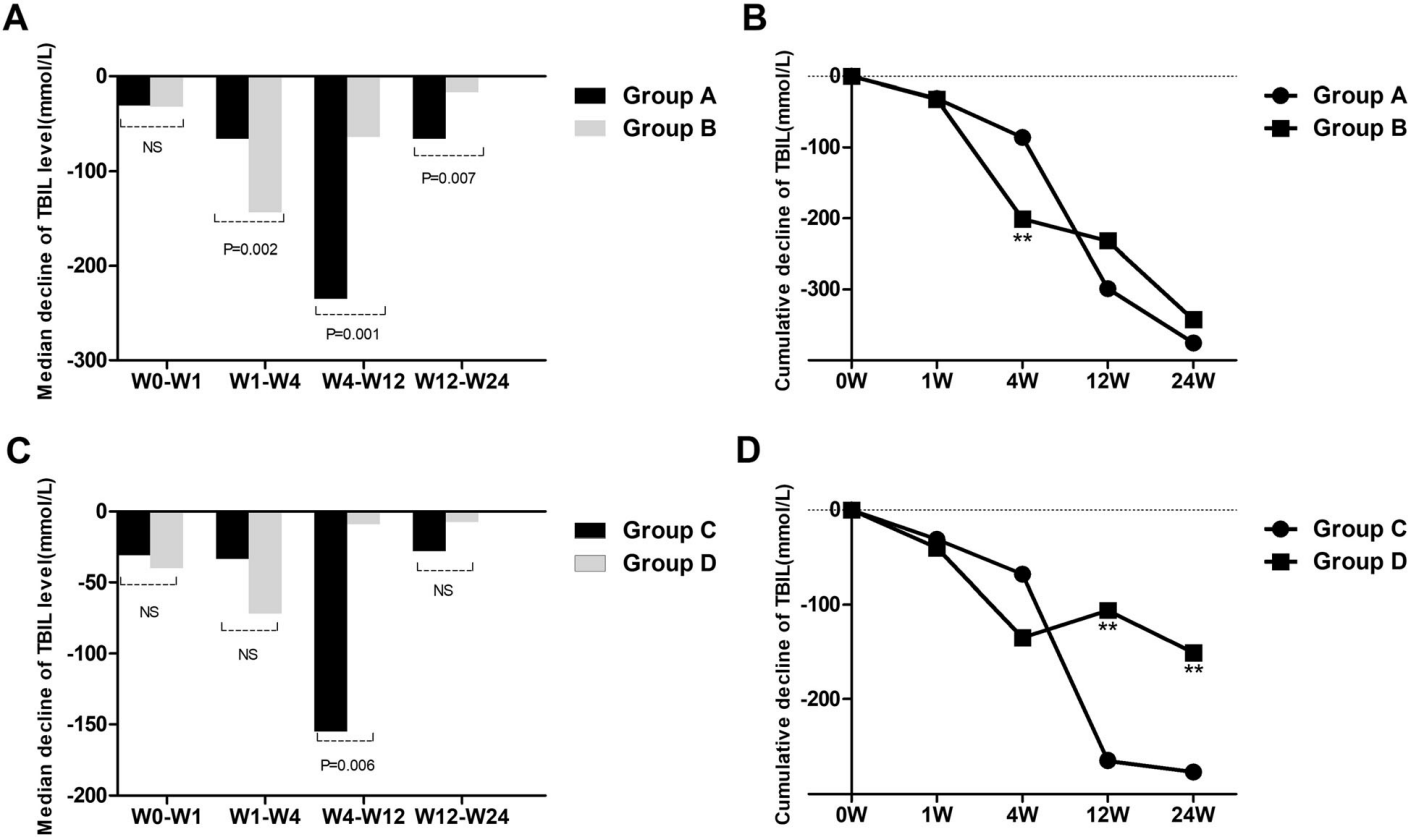
Comparison of the median decreases and cumulative decreases in TBIL levels between group A and group B and between group C and group D at different time points. **a** Median decrease in TBIL levels between group A and group B. **b** Cumulative decreases in TBIL levels between group A and group B. **c** Median decrease in TBIL levels between group C and group D. **d** Cumulative decreases in TBIL levels between group C and group D. Group A, liver failure patients treated with UCMSCs for more than 4 weeks (> 4 times); group B, liver failure patients treated with UCMSCs for less than or equal to 4 weeks (≤ 4 times). group C, liver cirrhosis patients treated with UCMSCs for more than 4 weeks (> 4 times); group D, liver cirrhosis patients treated with UCMSCs for less than or equal to 4 weeks (≤ 4 times). TBIL, total bilirubin. **P < 0.01

To understand whether the course of treatment affects the efficacy of UCMSCs, we compared the outcome of group A with that of group B in patients with liver failure. The median decrease in serum TBIL showed no difference between group A and group B at W0–W1 (Fig. 4a). The median decrease in serum TBIL in group A was significantly smaller than that in group B at W1–W4 (Fig. 4a). However, patients treated with UCMSCs for more than 4 weeks (group A) had larger decreases in TBIL levels than patients who received UCMSCs for less than or equal to 4 weeks (group B) at W4–W12 and W12–W24 (Fig. 4a). When the cumulative decrease in TBIL was compared between the two groups at different time points, no significant difference was found between the two groups at any observation week except week 4. The cumulative decrease in TBIL gradually increased in group A after 4 weeks of treatment, and no significant differences were maintained thereafter in either group (Fig. 4b). The decreases in ALT and AST of patients in group A were higher than those of patients in group B after 4 weeks of UCMSC treatment, and the PTA values and MELD scores of these groups differed before week 4 (Table 3).

**Table 3:** Comparative analysis of the therapeutic effect of UCMSCs in group A vs group B and group C vs group D at different time points

Outcome	Group	W0–W1		W1–W4		W4–W12		W12–W24	
		Value	***P*** value	Value	***P*** value	Value	***P*** value	Value	***P*** value
**ALT**	Group A	37.5 (5.25–214)	NS	5 (− 6 to 42)	NS	8 (− 1.25 to 23.5)	0.002	− 3 (− 11 to 5)	NS
	Group B	24 (9–260)		22.5 (6.2–45.3)		− 6 (− 15 to 6)		0.5 (− 4 to 5.5)	
	Group C	19 (2–48)	NS	− 0.5 (− 9.3 to 10.25)	0.041	2.5 (− 0.5 to 11)	0.014	− 4 (− 12 to 60)	NS
	Group D	12.5 (− 0.5 to 98.5)		9.5 (− 0.75 to 9.5)		− 6 (− 24.5 to 3.5)		5.5 (0.25–7)	
**AST**	Group A	17 (− 5.5 to 60.25)	NS	− 1 (− 17 to 11)	0.010	26 (6–53.5)	0.006	9 (− 5 to 20)	0.032
	Group B	24 (− 10 to 52)		16 (− 1 to 43.75)		1 (− 15 to 22)		3 (− 0.25 to 14.5)	
	Group C	13 (4–28)	NS	− 7.5 (− 16.75 to 17)	NS	26 (7.5–40.25)	0.000	− 3 (− 15 to 9)	NS
	Group D	4 (− 4 to 33.75)		7.5 (− 7 to 21.25)		4 (− 11.5 to 9)		8.5 (3.25–17)	
**PTA**	Group A	4 (− 1.75 to 6)	0.019	− 1 (− 7 to 3.5)	0.033	− 7 (− 15.5 to 0.5)	NS	− 3 (− 5.7 to 2.1)	NS
	Group B	− 2 (− 4 to 3)		− 5 (− 12 to − 1)		− 15 (− 22.5 to − 4.75)		− 4 (− 7.2 to 1.9)	
	Group C	5 (− 4 to 7)	0.014	− 1 (− 7 to 2.5)	0.008	− 7 (− 11.75 to 1.5)	NS	− 2 (− 4.1 to 1.9)	NS
	Group D	− 2 (− 8 to 1)		− 10 (− 19 to − 4)		− 4 (− 14.5 to 5)		− 3 (− 5.2 to 2.6)	
**MELD**	Group A	− 1 (− 2 to 1)	0.044	1 (− 1 to 2.5)	0.000	6.11 ± 5.32	NS	2.14 ± 1.71	NS
	Group B	1 (− 1 to 2)		4 (2–6)		6.86 ± 5.01		1.96 ± 1.64	
	Group C	0 (− 1 to 2)	NS	1 (− 1 to 3)	0.011	4 (0.5–7)	NS	2.4 (0.1–5.3)	NS
	Group D	2 (0–4)		4 (1–6.5)		0.5 (− 0.75 to 5.5)		3.3 (− 0.5 to 4.9)	

*NS* nonsignificant, *ALT* alanine aminotransferase, *AST* glutamic-oxaloacetic transaminase, *TBIL* total bilirubin, *PTA* prothrombin time activity, *MELD* model for end-stage liver disease

### Comparative analysis of the therapeutic effect of UCMSCs between group C and group D

Similarly, we compared the outcome of group C with that of group D in liver cirrhosis patients to understand the treatment course and the efficacy of UCMSCs. The median decrease in serum TBIL in patients treated with UCMSCs for more than 4 weeks (group C) was larger than that in group D at W4–W12 (Fig. 4c). The cumulative decrease in TBIL in group C gradually increased from week 4 and was larger than that of group D (Fig. 4d). Similarly, the decreases in ALT and AST in patients in group C were also larger than those in group D after 4 weeks of UCMSC treatment, and the PTA values and MELD scores of these two groups were significantly different before week 4 (Table 3).

### Comparative analysis of the therapeutic effect of UCMSCs between group A and group C

**Fig. 5:**
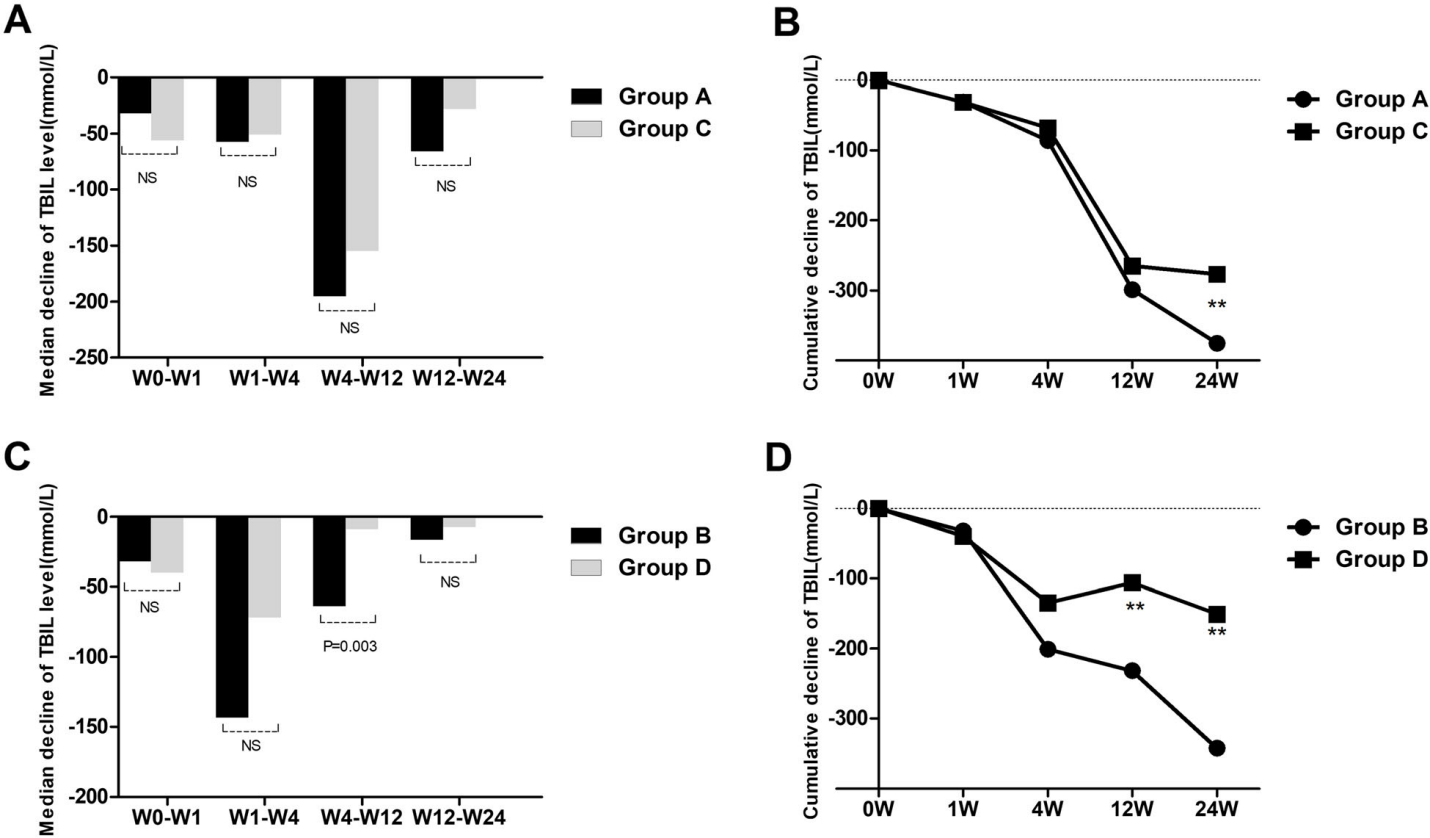
Comparison of the median decreases and cumulative decreases in TBIL levels between group A and group C and between group B and group D at different time points. **a** Median decrease in TBIL levels between group A and group C. **b** Cumulative decreases in TBIL levels between group A and group C. **c** Median decrease in TBIL levels between group B and group D. **d** Cumulative decreases in TBIL levels between group B and group D. Group A, liver failure patients treated with UCMSCs for more than 4 weeks (> 4 times); group C, liver cirrhosis patients treated with UCMSCs for more than 4 weeks (> 4 times); group B: liver failure patients treated with UCMSCs for less than or equal to 4 weeks (≤ 4 times); group D, liver cirrhosis patients treated with UCMSCs for less than or equal to 4 weeks (≤ 4 times). TBIL, total bilirubin. **P < 0.01

To investigate the therapeutic effect of UCMSC treatment for more than 4 weeks in patients with liver failure and patients with liver cirrhosis, we compared the outcome of group A with that of group C. The median decreases in serum TBIL, ALT, AST, and PTA values and MELD scores at all postbaseline time points were not significantly different between group A and group C (Fig. 5a) (Table 4). However, the cumulative decrease in TBIL of patients in group A was higher than that of patients in group C at week 24 (Fig. 5b).

**Table 4:** Comparative analysis of the therapeutic effect of UCMSCs in group A vs group C and group B vs group D at different time points

Outcome	Group	W0–W1		W1–W4		W4–W12		W12–W24	
		Value	***P*** value	Value	***P*** value	Value	***P*** value	Value	***P*** value
**ALT**	Group A	37.5 (5.25–214)	NS	5 (− 6 to 42)	NS	8 (− 1.25 to 23.5)	NS	− 3 (− 11 to 5)	NS
	Group C	19 (2–48)		− 0.5 (− 9.3 to 10.25)		2.5 (− 0.5 to 11)		− 4 (− 12 to 60)	
	Group B	24 (9–260)	0.042	22.5 (6.2–45.3)	NS	− 6 (− 15 to 6)	NS	0.5 (− 4 to 5.5)	NS
	Group D	12.5 (− 0.5 to 98.5)		9.5 (− 0.75 to 9.5)		− 6 (− 24.5 to 3.5)		5.5 (0.25–7)	
**AST**	Group A	17 (− 5.5 to 60.25)	NS	− 1 (− 17 to 11)	NS	26 (6–53.5)	NS	9 (− 5 to 20)	NS
	Group C	13 (4–28)		− 7.5 (− 16.75 to 17)		26 (7.5–40.25)		− 3 (− 15 to 9)	
	Group B	24 (− 10 to 52)	NS	16 (− 1 to 43.75)	NS	1 (− 15 to 22)	NS	3 (− 0.25 to 14.5)	NS
	Group D	4 (− 4 to 33.75)		7.5 (− 7 to 21.25)		4 (− 11.5 to 9)		8.5 (3.25–17)	
**PTA**	Group A	4 (− 1.75 to 6)	NS	− 1 (− 7 to 3.5)	NS	− 7 (− 15.5 to 0.5)	NS	− 3 (− 5.7 to 2.1)	NS
	Group C	5 (− 4 to 7)		− 1 (− 7 to 2.5)		− 7 (− 11.75 to 1.5)		− 2 (− 4.1 to 1.9)	
	Group B	− 2 (− 4 to 3)	NS	− 5 (− 12 to − 1)	NS	− 15 (− 22.5 to − 4.75)	NS	− 4 (− 7.2 to 1.9)	NS
	Group D	− 2 (− 8 to 1)		− 10 (− 19 to − 4)		− 4 (− 14.5 to 5)		− 3 (− 5.2 to 2.6)	
**MELD**	Group A	− 1 (− 2 to 1)	NS	1 (− 1 to 2.5)	NS	6.11 ± 5.32	NS	2.14 ± 1.71	NS
	Group C	0 (− 1 to 2)		1 (− 1 to 3)		4 (0.5–7)		2.4 (0.1–5.3)	
	Group B	1 (− 1 to 2)	NS	4 (2–6)	NS	6.86 ± 5.01	NS	1.96 ± 1.64	NS
	Group D	2 (0–4)		4 (1–6.5)		0.5 (− 0.75 to 5.5)		3.3 (− 0.5 to 4.9)	

*NS* nonsignificant, *ALT* alanine aminotransferase, *AST* glutamic-oxaloacetic transaminase, *TBIL* total bilirubin, *PTA* prothrombin time activity, *MELD* model for end-stage liver disease

### Comparative analysis of the therapeutic effect of UCMSCs between group B and group D

The outcomes of patients with liver failure who received UCMSCs for less than or equal to 4 weeks in group B were compared with those of patients with liver cirrhosis in group D to learn more about the therapeutic effect of UCMSCs. The median decrease in TBIL was not different between the two groups at any observation week except weeks 4–12 (Fig. 5c). At weeks 4–12, the median decrease in TBIL in group B was larger than that in group D (*P* = 0.003, Fig. 5c). The cumulative decrease in TBIL of patients in group B was larger than that of group D at week 12 and week 24 (Fig. 5d). The levels of ALT, AST, and PTA and the MELD scores at all postbaseline time points were not significantly different between group B and group D (Table 4).

## Discussion

Although the gold standard therapy for HBV-related end-stage liver disease is liver transplantation, there are discrepancies between liver supply and liver demand. Stem cell therapy is an alternative approach to liver transplantation for HBV-related liver failure and liver cirrhosis. Previous research from our department shows that allogeneic bone marrow-derived MSCs are safe and effective for patients with HBV-related ACLF [8, 9]. However, ethical issues, uncertainty about malignant differentiation in vivo, and the need for culture limit its application and clinical significance. UCMSCs have shown great potential in regenerative medicine due to their abundant sources, multilineage differentiation potential, low immunogenicity, and self-renewal ability [15]. In the CCl_4_-induced acute liver injury model, significant hepatoprotective effects of UCMSCs were observed, with decreased levels of hepatocellular necrosis and lobular neutrophilic infiltration [16]. A recent study [17] found that UCMSC treatment can disrupt the inflammatory cascade by inhibiting monocyte activation, and peripheral infusion of human UCMSCs rescues acute liver failure lethality in monkeys. In this study, UCMSCs were used to treat patients with HBV-related liver failure. We found that the levels of ALT, AST, and TBIL and MELD scores gradually decreased and that PTA values gradually increased after UCMSC treatment. This finding is consistent with previous reports showing that UCMSC transfusions significantly increased survival rates in patients with ACLF; reduced MELD scores; increased serum albumin, cholinesterase, and prothrombin activity; and increased platelet counts [7]. Yan et al.’s study [18] demonstrated that UCMSCs could deliver GPX1 to exert hepatocyte protective effects by detoxifying CCl_4_ and H_2_O_2_ and reducing oxidative stress and apoptosis in hepatocytes. miR-455-3p-enriched exosomes derived from UCMSCs could attenuate macrophage infiltration and local liver damage and reduce the serum levels of inflammatory factors, thereby improving liver histology and systemic disorders [19]. When MSCs were used to treat liver disease, the transdifferentiation of MSCs into hepatocytes was observed, and MSCs also secreted various bioactive molecules to promote liver regeneration [20]. Therefore, it was not unexpected that UCMSCs would have such a good therapeutic effect on liver failure in this study.

The characteristics of MSCs include continuous self-renewal, proliferation, multipotent differentiation, and immunomodulatory activities. UCMSCs possess not only the common characteristics of MSCs but also more stable biological characteristics, relatively easy accessibility, an abundant source, and no ethical issues, making UCMSCs a good choice for the treatment of liver fibrosis [21]. In this study, the therapeutic effect of UCMSCs on liver cirrhosis was also investigated. Our results reveal that treatment with MSCs significantly improves liver function in patients with liver cirrhosis, as evidenced by the levels of ALT, AST, and total bilirubin. However, it has no beneficial effects in terms of PTA values and MELD scores. This finding is consistent with previous systematic reviews and meta-analyses of the therapeutic efficacy of MSCs against liver disease [22]. In addition, ascites in patients with decompensated liver cirrhosis could be improved by UCMSCs [6]. In a CCl_4_-induced rat liver fibrosis model [23], UCMSCs could differentiate into functional hepatocytes that improved both biochemical and histopathologic changes. In addition, secretomes from UCMSCs also reduced the liver expression of multiple fibrotic factors, collagens, metalloproteinases, TGFβ, and smad proteins in the TGFβ signaling pathway [24]. Furthermore, UCMSCs also have a significant immune modulatory effect on immune cells [25]. Therefore, UCMSCs can be a simple and effective treatment for the management of fibrotic liver diseases.

The dosage and duration of MSC treatment are two important issues. Using too many intraportal or intrahepatic BM-MNCs is counterproductive, and it seems more beneficial when they are enriched to reduce the cell number [11]. In this study, we mainly focused on whether prolonging the treatment course can improve the curative effect of UCMSCs. Before 4 weeks of UCMSC treatment, the median decrease and cumulative decrease in TBIL were not significantly different between patients with prolonged treatment and patients with a standard 4-week treatment course. Moreover, the median decrease and cumulative decrease in TBIL of liver patients with a standard 4-week treatment course were larger than those of liver patients with prolonged treatment. Surprisingly, the data indicate that the median decrease and cumulative decrease in TBIL of patients with prolonged treatment were equivalent to or even surpassed the decreases of patients with a standard 4-week treatment course. Our results show that extending the treatment course may be an option to improve the efficacy of UCMSCs. At present, few clinical trials have investigated the relationship between efficacy and MSC number. A recent meta-analysis [26] suggested that the number of cells injected was an important factor influencing the efficacy of autologous MSC therapy, and a dose of > 4 × 10^8^ BMSCs was more beneficial for patients. However, some clinical trials with controls using adult stem cells showed that cell number might play a role in the result and that too many cells might be deleterious [11]. Our results show that increasing the dosage of MSCs by extending the course of treatment may be one of the most appropriate methods of using UCMSCs to treat liver disease. In the future, large-scale and prospective studies are required to identify prolonged strategies for patients receiving UCMSC therapies.

The last issue we are concerned about is whether UCMSCs are more suitable for liver failure or liver cirrhosis. The results of this study showed that only the median TBIL decrease of patients with liver failure was larger than that of patients with liver cirrhosis after 4 weeks of UCMSC treatment; no statistically significant differences in the levels of ALT or AST, the PTA values or the MELD scores were found between patients with liver failure and patients with liver cirrhosis at all observation weeks. However, the median decrease and cumulative decrease in the TBIL level of patients with liver failure who received a standard 4-week treatment course were larger than those of patients with liver cirrhosis. Pseudo lobule formation and destruction of the liver parenchyma may limit the transdifferentiation of UCMSCs into hepatocytes. In addition, liver sinusoidal endothelial cells (LSECs) may play an important role in this difference. LSECs are the first cells affected by liver or chest injury and are involved in the response of the liver to damage. LSECs govern initiation of the regenerative process initiation, and aberrant LSEC activation in chronic liver injury induces fibrosis. Sustaining hepatic injury can lead to loss of the LSEC phenotype and protective properties, promote angiogenesis and vasoconstriction, and contribute to inflammation and fibrosis [27]. Shubham et al. [28] found that cellular and functional loss of liver endothelial cells correlates with poor hepatocyte regeneration in ACLF. Therefore, a standard 4-week UCMSC treatment for liver cirrhosis failed to achieve the same satisfactory effect as treatment for liver failure; strategies for prolonging treatment for patients with liver cirrhosis may be an option after weighing the advantages and disadvantages.

There were some limitations of this study. First, this study was a retrospective study. Second, this study was based on data from only one health center, yielding a rather small cohort and possible examiner bias. Third, the study period was only 24 weeks; a longer study period could provide additional insights. In addition, the number of patients evaluated for treatment efficacy was relatively small. In the future, large-scale and prospective studies are required to confirm the therapeutic effect of UCMSCs in HBV-related end-stage liver disease.

In conclusion, peripheral infusion of UCMSCs showed good therapeutic effects for HBV-related liver failure and liver cirrhosis; prolonging the treatment course can increase the curative effect of UCMSCs for end-stage liver disease, especially for patients with cirrhosis. The long-term efficacy of this approach should be further assessed in a randomized, large-scale, double-blind, and well-controlled trial.

## Data Availability

The data that support the findings of this study are available from the corresponding author upon reasonable request.

## Abbreviations

HBV: Hepatitis B virus
ALT: Alanine aminotransferase
UCMSCs: Umbilical cord mesenchymal stem cells
ACLF: Acute-on-chronic liver failure
TBIL: Total bilirubin
PTA: Prothrombin activity
MELD: Model for end-stage liver disease
